# Case of Dislocation of the Spine

**Published:** 1814-04

**Authors:** John Bond

**Affiliations:** Surgeon


### *For the Medical and Physical Journal.*

### *Case of Dislocation of the Spine; by* Mr. John Bond, Surgeon.

AS it is the opinion of most medical authors, that dislocations of the spine never occur without fracture, in which I have hitherto coincided, but there being no reason to suspect fracture in the following case, I cannot help considering it a very unfrequent occurrence, which induces me to wish it inserted in your Journal.

William Thorn, the subject of the following case, was working in a stone pit in a delving position, when a considerable weight of stone fell upon him, and crushed him to the ground, in which state he was discovered.

When released, he walked betwixt two and three hundred yards with great difficulty, and nearly double, from whence he was conveyed home in a cart, and the next morning I saw him, when the following symptoms presented themselves. He had completely lost the use of the lower limbs, and also the power of speech; secretions and excretions were totally suppressed from the moment of the accident; pulse depressed, and under 60.

These appearances, connected with the nature of the accident, led me of course to suspect injury being done to the spine, and on turning him upon his face, my suspicions were soon verified.

There was an evident depression in the spine, occasioned by a partial dislocation of the fourth dorsal vertebra, which I immediately attempted to remove by placing my feet upon his shoulders, and pulling at his head, which I kept during my exertions to incline a little towards the sternum, so as to make the part where the injury was sustained an obtuse angle with the chin and pelvis. His pulse instantly rose in fullness and frequency; his pain (as he afterwards informed me) was much lessened; but no further amendment appeared to be derived at the time, and I left him with a full persuasion that nothing more could be done. Towards night he was considered much worse by his friends, and at two o'clock in the morning was thought to be dying, when his next neighbour was sent for to assist in changing his position, and placing both hands behind his head for that purpose, assisted by another at his feet, in making the attempt, made the angle before described more acute, and the dislocation was instantaneously though not completely reduced. The lower limbs were almost immediately restored to their sensibility and use; his speech he so instantly recovered as to inquire of the people present "didn't you hear it crack." The sensation he described afterwards, and it appeared to me to resemble exactly a dislocated humerus, when it goes with force into its socket. The bladder and intestines very soon performed their functions; notwithstanding which a good deal of pain remained in the part, accompanied with a complete inability to turn his head either to the right or left, until I had made a second attempt similar to the first in the course of that day, when the parts were restored to their accustomed ease and action; and in a few days he walked to my house, a distance of five miles.

*Nuneaton*, JOHN BOND.

*Feb.* 9, 1814.

